# Whole-Genome Sequence of the Bacteriophage-Sensitive Strain *Campylobacter jejuni* NCTC12662

**DOI:** 10.1128/genomeA.00409-17

**Published:** 2017-05-25

**Authors:** Yilmaz Emre Gencay, Martine C. H. Sørensen, Lone Brøndsted

**Affiliations:** Department of Veterinary and Animal Sciences, University of Copenhagen, Frederiksberg, Denmark

## Abstract

*Campylobacter jejuni* NCTC12662 has been the choice bacteriophage isolation strain due to its susceptibility to *C. jejuni* bacteriophages. This trait makes it a good candidate for studying bacteriophage–host interactions. We report here the whole-genome sequence of NCTC12662, allowing future elucidation of the molecular mechanisms of phage–host interactions in *C. jejuni*.

## GENOME ANNOUNCEMENT

*Campylobacter jejuni* is a zoonotic Gram-negative bacterium and the leading cause of foodborne gastroenteritis in the western world. Research within bacteriophages as biocontrols of *C. jejuni* has resulted in the isolation of phages belonging to two genera: *Cp220virus* and *Cp8virus*. *C. jejuni* NCTC12662 is susceptible to most of these phages, including phages dependent on the capsular polysaccharide (CPS) or a motile flagellum for infection (1). Here, we report the genome sequence of NCTC12662 as a common host for *C. jejuni* phages, allowing future molecular investigation and comparison of phage–host interactions in *C. jejuni*.

DNA libraries from the genomic DNA from *C. jejuni* NCTC12662 (obtained from the National Collection of Type Cultures) were prepared using the Nextera XT version 3 kit (Illumina) and sequenced with MiSeq (Illumina) in 2 × 250-bp operating mode. The 550,920 reads generated were *de novo* assembled using CLC Genomics Workbench version 9.5.3, resulting in a total of 47 contigs. Due to the obtained low coverage, another round of sequencing was executed at the Sanger Institute (Cambridge, United Kingdom) using HiSeq 2000 (Illumina), yielding 4,635,024 100-bp raw reads. Subsequently, contigs were joined by the extend and align contig functions using flanking genome data, resulting in an average coverage of 287-fold. The genome sequence was annotated using the NCBI Prokaryotic Genome Annotation Pipeline (2). The circular genome of NCTC12662 is 1,612,586 bp with an average G+C content of 30.7%. It comprises 1,548 coding sequences, 44 tRNA genes, and 3 rRNA operons and carries no prophage-associated genes (3) or plasmids.

Phase-variable expression of genes that carry polyG tracts is a well-known phenomenon for generating phenotypic population diversity in *C. jejuni* (4), and *C. jejuni* NCTC12662 contains 19 polyG tracts. Previously, we found that the phase-variable expression of *cj1421* and *cj1422*, which modify Gal*f*Nac and heptose residues of the CPS with *O*-methyl phosphoramidate (MeO*P*N), respectively, were responsible for phage resistance in *C. jejuni* NCTC11168 (5, 6). NCTC12662 encodes one putative phase-variable MeO*P*N-transferase (*06810*), showing 83% identity to Cj1421 and Cj1422 at the N-terminal part of the protein. Although this indicates that a different CPS residue of NCTC12662 is modified by MeO*P*N compared to NCTC11168, the phase-variable nature of gene *06810* is conserved in NCTC12662.

NCTC12662 harbors 7 clustered regularly interspaced palindromic repeats (CRISPR) with subtype-II-C CRISPR-associated genes (Cas-1, -2, and -9) sharing high similarity with NCTC11168. However, one of the protospacers is duplicated, and thus NCTC12662 encodes only 5 distinct 31-bp protospacers. Noteworthy, the duplicate protospacer exclusively matches a hypothetical protein (CJE0597) found in *C. jejuni* integrated element 2 (CJIE2) in strain RM1221 (7) and group 4 prophages CJIE4-1 and CJIE4-5 (8). Whether the absence of both groups of prophages in NCTC12662 is due to CRISPR-Cas activity is an intriguing question. Sequence homologies also suggest that NCTC12662 encodes type I, II, III, and IV RM systems (9), indicating no significant effect of these RM systems in *C. jejuni* phage resistance. Thus, further studies are needed to elucidate why NCTC12662 is susceptible to many diverse phages of *C. jejuni*.

### Accession number(s).

The *C. jejuni* NCTC12662 complete genome is available under GenBank accession no. CP019965
